# Recent Developments in Functional Polymers via the Kabachnik–Fields Reaction: The State of the Art

**DOI:** 10.3390/molecules29030727

**Published:** 2024-02-04

**Authors:** Rui Yuan, Xianzhe He, Chongyu Zhu, Lei Tao

**Affiliations:** 1 The Key Laboratory of Bioorganic Phosphorus Chemistry and Chemical Biology (Ministry of Education), Department of Chemistry, Tsinghua University, Beijing 100084, China; yr22@mails.tsinghua.edu.cn (R.Y.); hexz19@mails.tsinghua.edu.cn (X.H.); 2 Key Lab of Science and Technology of Eco-Textile, Ministry of Education, College of Chemistry and Chemical Engineering, Donghua University, Shanghai 201620, China; czhu@dhu.edu.cn

**Keywords:** Kabachnik–Fields reaction, functional polymer, polymer chemistry

## Abstract

Recently, multicomponent reactions (MCRs) have attracted much attention in polymer synthesis. As one of the most well-known MCRs, the Kabachnik–Fields (KF) reaction has been widely used in the development of new functional polymers. The KF reaction can efficiently introduce functional groups into polymer structures; thus, polymers prepared via the KF reaction have unique α-aminophosphonates and show important bioactivity, metal chelating abilities, and flame-retardant properties. In this mini-review, we mainly summarize the latest advances in the KF reaction to synthesize functional polymers for the preparation of heavy metal adsorbents, multifunctional hydrogels, flame retardants, and bioimaging probes. We also discuss some emerging applications of functional polymers prepared by means of the KF reaction. Finally, we put forward our perspectives on the further development of the KF reaction in polymer chemistry.

## 1. Introduction

Multicomponent reactions (MCRs) are modular and efficient reactions using at least three different reactants to synthesize a single complex functional product in a reactor, with negligible by-products. Recently, many MCRs have been widely used in the development of new functional polymers. These MCRs include the Passerini [1,2], Ugi [3,4], Biginelli [5,6,7], Kabachnik–Fields [8,9], Hantzsch [10,11], and Mannich reactions [12,13] as well as thiolactone-based [14] and metal-catalyzed multicomponent reactions [15]. The functional polymers prepared by means of these MCRs have been further applied in the fields of environmental science [16,17], material science [18,19], and biomedicine [20].

**Figure 1:**
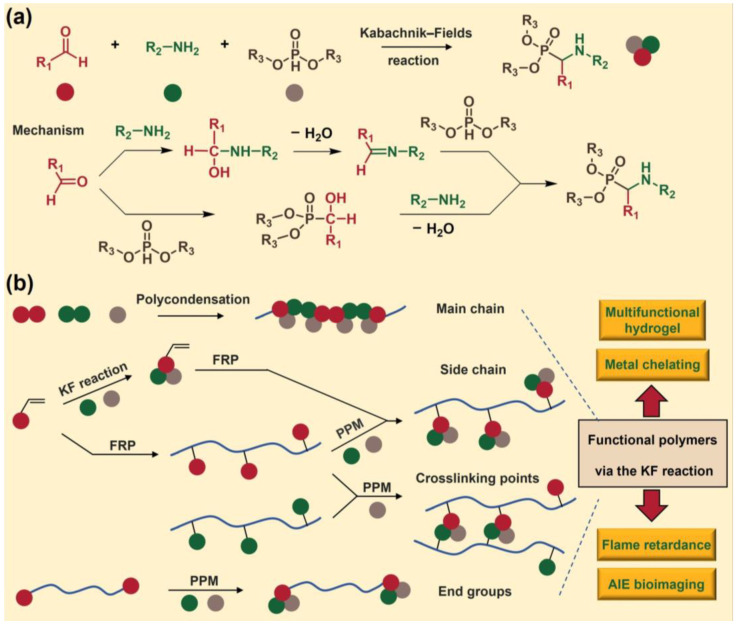
(**a**) Mechanism of the KF reaction. (**b**) Scheme of the KF reaction synthesizing functional polymers for multiple applications.

Among these MCRs, the KF reaction, introduced by M. I. Kabachnik and E. K. Fields in 1952, is one of the most famous reactions [21]. It is a three-component reaction that produces an α-aminophosphonate structure through common reactants (aldehydes, amines, and phosphites) (Figure 1a). There are two possible reaction mechanisms for the Kabachnik–-Fields reaction [22]. The first is that the aldehyde first reacts with the primary amine to form an imine (Schiff base) intermediate, and then the phosphite is added to the imine structure through a Pudovik reaction to prepare an α-aminophosphonate compound. The second possible reaction mechanism is that the phosphite diester is first added to aldehyde through an Abramov reaction to form an α-hydroxyphosphonate structure, which is then replaced by an amine to prepare the target α-aminophosphonate compound. This reaction has a high efficiency under mild reaction conditions, and water is its only by-product. Meanwhile, the α-aminophosphonate structure produced by the KF reaction exhibits many important biological activities and has been widely studied in the fields of organic chemistry, pharmacy, and biology [23,24]. In addition, the structure has been proven to have potential metal chelating abilities and flame retardancy properties [8,25,26,27].

In the past, polymer chemists focused on the development of a polymer synthesis methodology based on the KF reaction, resulting in a series of KF reaction-based polymers through polycondensation, free radical polymerization (FRP), and post-polymerization modification (PPM) [28,29,30,31] (Figure 1b). The dialdehydes, diamines, and phosphites that can be used for polycondensation through the KF reaction are easily available. The monomers containing α-aminophosphonate pendent groups for free radical polymerization (FRP) can be easily prepared through efficient KF reactions using polymerizable aldehydes or amines; then, functional polymers can be simply obtained using these monomers through FRP. Meanwhile, polymerizable aldehydes or amines can also be polymerized to obtain poly(amines) or poly(aldehydes) in advance. These polymers can be post-modified through the KF reaction to develop new functional polymers with pendent α-aminophosphonate groups. Additionally, functional polymers containing α-aminophosphonate structures as the end groups or crosslinking points can also be developed through the PPM strategy. These studies provide a solid foundation for the further development of the KF reaction in polymer science.

Recently, the KF reaction has been employed in the development of new functional polymers due to its unique functional structure and efficient coupling of multicomponents. The KF reaction has a significant importance in the preparation of new functional polymers. The KF reaction uses more than two substrates to produce α-aminophosphonate. Thus, the KF reaction can be used to introduce different substrates with specific functional groups into polymer chains to achieve multifunctional polymers. Meanwhile, the unique α-aminophosphonate structures produced by the KF reaction have metal chelating abilities, flame retardant properties, and some biological activities. Polymers with these new functions may be developed by including α-aminophosphonate structures in the main chains or side chains through the KF reaction. In this mini-review, we summarize the latest progress of the KF reaction in the development of functional polymers for heavy metal adsorbents, multifunctional hydrogels, flame retardants, and bioimaging probes. We also discuss some emerging applications of the functional polymers prepared by the KF reaction. Finally, we put forward our perspectives on the further development of the KF reaction in polymer chemistry.

## 2. Heavy Metal Adsorbents

With the development of new energy sources and high-tech electronics, heavy metals such as Cu, Hg, Zn, Cr, Cd, and rare earth metals have been irreplaceable in modern society. However, the heavy metal ions generated during the production process may invade the water cycle and even enter the human body through a series of pathways, causing great harm to the environment and human health [32,33,34,35]. In particular, the accumulation of heavy metals will increase many health risks such as carcinogenic, mutagenic, and teratogenic risks, presenting a deleterious threat to living organisms [36]. Ever since the first appearance of Minamata disease in Japan in 1956, the effective removal of heavy metal ions has become a global hot topic.

**Figure 2:**
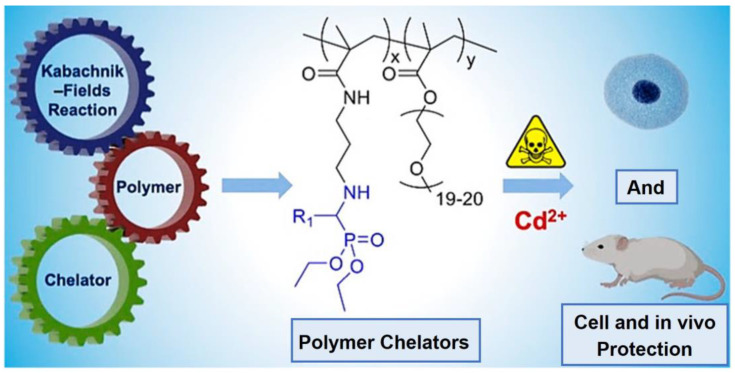
Exploration of safe and efficient polymer chelators by combining the KF reaction, chelator, and polymer chemistry for preventing acute and chronic heavy metal poisoning in mice. Reprinted with permission from ref. [8]. Copyright 2022 American Chemical Society. All rights reserved.

In recent decades, polymer-based heavy metal adsorption materials have drawn increasing intention owing to their good stability, high chelating efficiency, and easy separation after ion capture [37,38,39]. Among them, polymers that can be easily prepared via the KF reaction have proven to be excellent candidates for heavy metal chelating. Similar to many phosphonate ligands, the unique α-aminophosphonate structure produced by the KF reaction is able to direct complex metal ions with a high efficiency [40]. In 2020, Tao et al. prepared a series of polymers through the KF reaction, introducing an α-aminophosphonate structure into the polymer backbone (Figure 2), and demonstrated their potential as polymer chelating agents against heavy metal poisoning [8].

**Figure 3:**
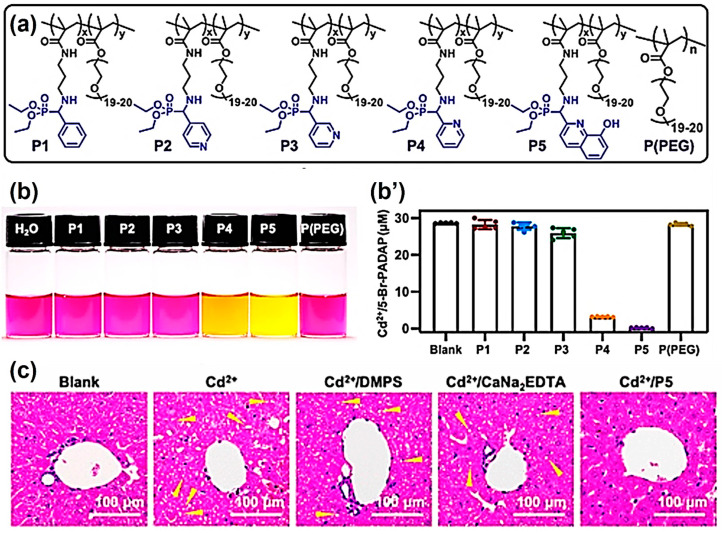
(**a**) Polymers used in the Cd^2+^ chelating tests. (**b**) Color change and (**b’**) concentrations of Cd^2+^/5-Br-PADAP (absorbance at 520 nm) in the presence of different polymers. Water serves as a blank. The data are presented as the mean ± SD (n = 5). (**c**) H&E staining of the liver tissues of mice after different treatments. Yellow arrows: hepatocyte steatosis. Reprinted with permission from ref. [8]. Copyright 2022 American Chemical Society. All rights reserved.

In this research, Cd^2+^ was used as a representative heavy metal ion. Various monomers containing different α-aminophosphonate structures were effectively synthesized by the KF reaction. These monomers were copolymerized with poly (ethylene glycol methyl ether) methacrylate (PEGMA) to prepare water-soluble polymers with excellent biosafety and a long metabolic life (Figure 3a). The Cd^2+^ chelating ability of these polymers was then screened with the aid of 2-(5-bromo-2-pyridylazo)-5-(diethylamino)phenol (5-Br-PADAP), a color indicator of free Cd^2+^ ions. In simple terms, the solution’s color changed from pink to yellow, suggesting an efficient complexing of Cd^2+^ by the KF polymers (Figure 3b). By measuring the UV absorbance at 520 nm, the Cd^2+^ scavenging ability of these polymers was quantified, and the polymer P5 exhibited the best Cd^2+^ scavenging performance (Figure 3b’). Then, the authors evaluated the in vivo Cd^2+^ protection of polymer P5. As a result, the Cd^2+^-poisoned mice injected with polymer P5 maintained good liver tissue conditions, like the healthy mice, while the livers of other Cd^2+^-poisoned mice treated with other small molecular chelating agents still showed different levels of damages (Figure 3c). These results indicated that polymers prepared by KF reaction have excellent heavy metal scavenging abilities and the potential for the in vivo protection of acute or chronic Cd^2+^ poisoning in organisms.

**Figure 4:**
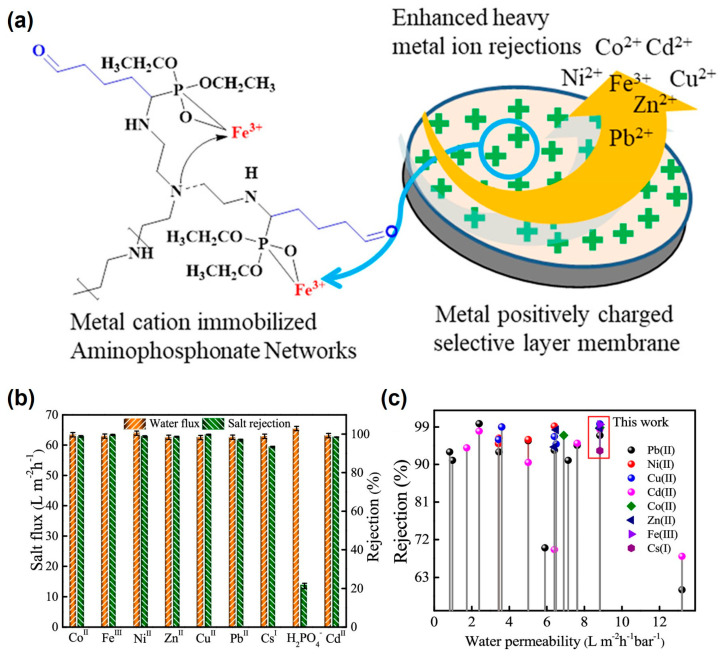
(**a**) Metal cation positively charges selective layer membranes for heavy metal ion rejection. (**b**) Rejection performance of heavy metal ions by the membranes. (**c**) Comparative salt retention performance with the existing literature. Testing conditions: salt solutions concentration = 1.0 g L^−1^, mixed salt solutions concentration, i.e., Co^II^/Zn^II^ = 0.5/0.5 g L^−1^, 21 °C, pH = 3.25, 0.8 MPa. Error bars are the standard deviations of three repeat experiments. Reprinted with permission from ref. [41]. Copyright © 2019 American Chemical Society. All rights reserved.

The efficient removal of heavy metal ions in water is also an important method of protecting the environment and organisms from heavy metal hazards. Polymer materials prepared by the KF reaction provide many new strategies for this purpose. In 2019, Liu et al. proposed an approach for fabricating α-aminophosphonate containing nanofiltration (NF) membranes with a metal–organic coordination in selective layers (Figure 4a) [41]. Through the KF reaction, they successfully transformed polyethyleneimine (PEI) into aminophosphonate polymers. The composite NF membranes were obtained by depositing a thin layer of the aminophosphonate polymers via an inorganic metal cation integration strategy. The aminophosphate polymer networks deposited on the NF membranes were coordinated and chelated with multivalent metal ions through stronger polar-covalent interactions not only based on electrostatic interactions of anionic charges. The NF membranes gave a high water flux (8.83 L m^−2^ h^−1^ bar^−1^) and an excellent rejection performance in efficiently intercepting and separating various heavy metal ions in water (Figure 4b). Compared with the data in the existing literature, the membrane prepared in this work exhibited a top performance in water flux and heavy metal ion rejection (Figure 4c). This work shows the potential of KF polymers applied in membranes for water treatment of heavy metal pollution, and it also provides a new idea regarding the modification of membranes for removing heavy metal ions in sewage.

**Figure 5:**
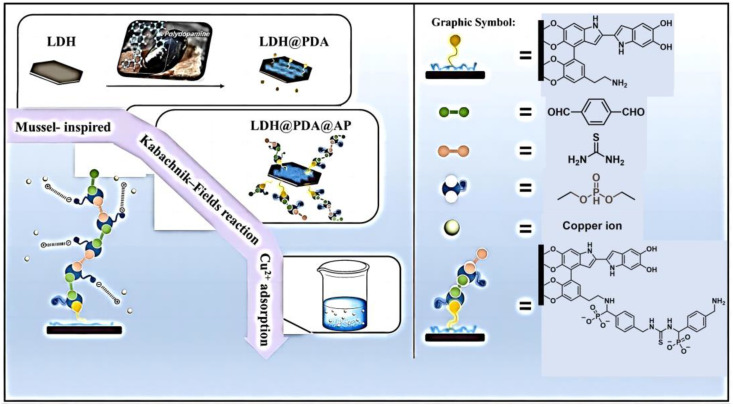
Preparation of mussel-inspired LDH through the modification of the functional polymer prepared by the KF reaction for highly efficient adsorption. Reprinted with permission from ref. [42]. Copyright © 2019 Elsevier Ltd. All rights reserved.

In addition to the modification of membranes, polymers prepared by the KF reaction have been also used to modify layered materials with high specific surface areas for the efficient removal of heavy metal ions. In 2019, Wei et al. proposed a method for the preparation of polymer-functionalized layered double hydroxides (LDH) to highly remove Cu^2+^ (Figure 5) [42]. This method anchored polydopamine and α-aminophosphonate chains on the original LDH nanosheets through a combination of the mussel-inspired chemistry and KF reaction. The modified LDH showed an excellent adsorption capacity of Cu^2+^, and the maximum adsorption capacity was calculated to be 105.44 mg g^−1^, which was impressive in the fields of LDH adsorbents. The kinetics and thermodynamic adsorption process of the modified LDH to Cu^2+^ was further discussed, which proved the endotherm and spontaneity of the adsorption process. This work not only achieves the surface modification of LDH-based materials through effective and general combination methods but also provides a new way to construct various multifunctional hybrid materials for the efficient removal of heavy metal ions.

**Figure 6:**
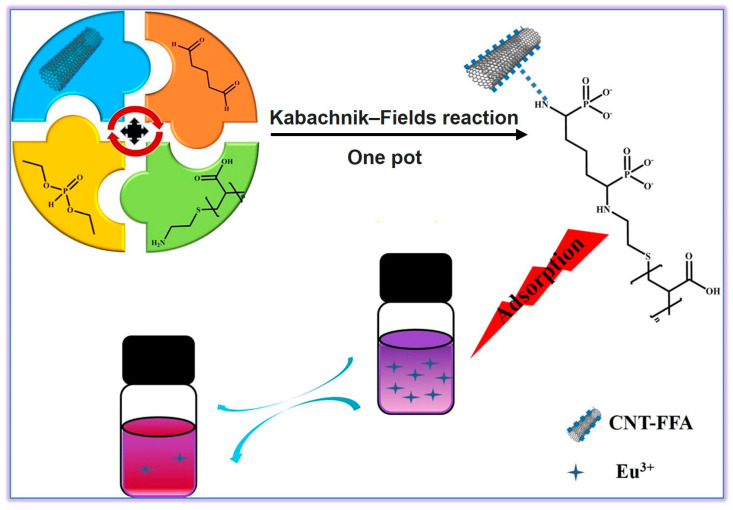
Fabrication of CNT-based polymer composites through DA cycloaddition and KF reactions for the removal of Eu^3+^ ions from wastewater. Reprinted with permission from ref. [46]. Copyright 2020 Elsevier B.V. All rights reserved.

Rare earth metals with unique optical and electrical properties have attracted extensive attention from researchers around the world, yet the enrichment and recovery of these precious resources from aqueous system remains challenging [43,44,45]. In 2020, Zhang et al. developed a novel strategy combining the Diels–Alder (DA) and the KF reactions for the preparation of carbon nanotubes (CNT)-based polymer composites for the removal of rare earth ions from wastewater (Figure 6) [46]. They introduced the amino groups of furfuryl amines into the CNT via the DA reaction and successfully synthesized the composites with α-aminophosphonate structures through the KF reaction in one pot. The fabricated composites (CNT-FFA-PAA) had a strong adsorption capacity for rare earth metal ions, with great potential to be used as adsorbents for recovering rare earth metal ions (Eu^3+^) in wastewater. Furthermore, this study also discussed the effects of a series of factors such as pH value, temperature, time, and initial concentration on the adsorption behavior of CNT-FFA-PAA and proved that the adsorption behavior of CNT-FFA-PAA towards rare earth ions can be adjusted through these factors. This work combines the KF reaction and the DA reaction for the first time for the surface modification of CNT, indicating the possibility of using polymers prepared via the KF reaction for further applications in the treatment and recovery of rare earth metals.

## 3. Multifunctional Hydrogels

**Figure 7:**
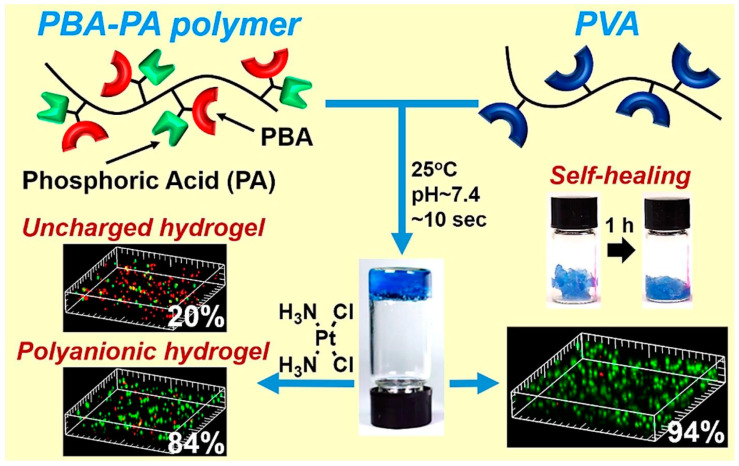
A polyanion self-healing hydrogel for the controlled release of cisplatin in a 3D cell culture through the KF reaction. Reprinted with permission from ref. [47]. Copyright 2020 Elsevier Ltd. All rights reserved.

Hydrogels are important biocompatible materials for cell cultures and medical uses. The introduction of the α-aminophosphonate structure into these hydrogels endows them with multifunctions for meeting different applications. In 2020, Tao et al. developed a biocompatible polyanion self-healing hydrogel for the controlled release of cisplatin by the KF reaction (Figure 7) [47]. Concretely, they prepared a multifunctional polymer-containing phenylboronic acid (PBA) and phosphonate (PA) groups by means of a one-step KF reaction and FRP, followed by an effective hydrolysis reaction. The negatively charged polymer was mixed with polyvinyl alcohol (PVA) to form a polyanion self-healing hydrogel under mild conditions (25 °C, pH = 7.4) through dynamic borate ester bonds. Due to the dynamics of the borate ester bonds, the hydrogel had a good self-healing ability. The strength and crosslinking network density of the hydrogel were adjustable by changing the amount of the polymer. The negatively charged PA groups in the α-aminophosphonate structures were combined with the positively charged cis-dichlorodiammineplatinum (II) (CDDP). Through an electrostatic interaction, the anticancer drug CDDP was gradually released in the control group. The hydrogel had a low cytotoxicity and a good biocompatibility, with great potential in biological and medical applications for the controlled release of positively charged drugs to treat the related diseases. This research led to the development of KF reactions in smart materials, prompting broader studies on multicomponent reactions in polymer chemistry and material science.

**Figure 8:**
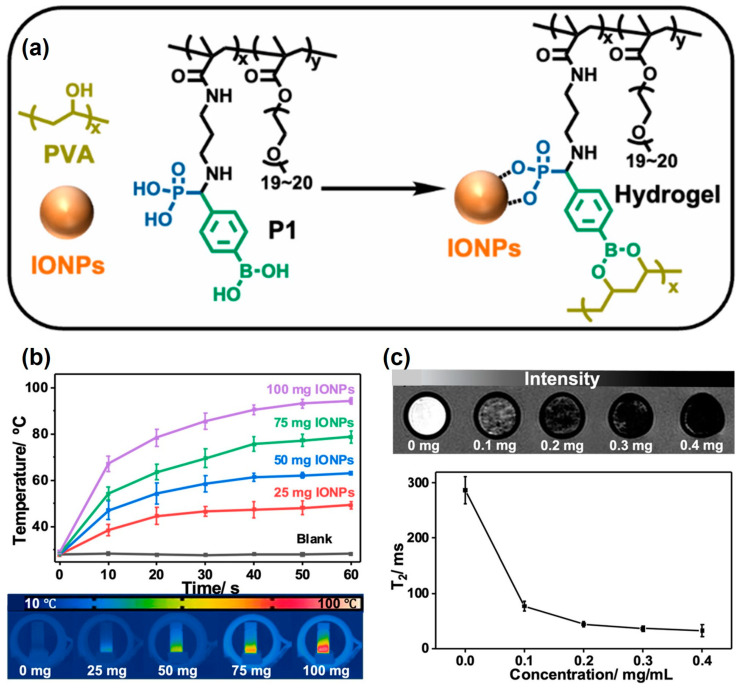
(**a**) Preparation of P1 and the P1–IONPs complex. (**b**) Temperature of P1–PVA hydrogels containing different amounts of IONPs vs. time in an AMF (f = 285 kHz, H = 201.2 Oe). Data are presented as the mean ± SD, n = 5. (**c**) MRI images and T_2_ values of P1–PVA hydrogels containing different amounts of IONPs. Reprinted with permission from ref. [9]. Copyright 2022 American Chemical Society. All rights reserved.

Besides electrostatic interactions, the metal chelating ability of the α-aminophosphonate structures produced by the KF reaction also enriches the application of hydrogels. In 2022, Tao et al. reported a magnetic self-healing hydrogel produced via the KF reaction [9]. To prepare the hydrogel, a polymer (P1) containing both phenylboronic acid (PBA) and phosphonic acid (PA) groups was synthesized by means of the KF reaction (Figure 8a). The PA groups in the α-aminophosphonate structures dispersed iron oxide nanoparticles (IONPs) through strong interactions, and the PBA groups rapidly reacted with the PVA to form a self-healing hydrogel containing well-dispersed IONPs. The hydrogel exhibited good self-healing ability and injectability owing to the dynamic borate ester network. The magnetic hydrogel was able to absorb energy from alternating magnetic fields (AMFs) and convert it into heat, showing good magnetothermal properties (Figure 8b). Additionally, it reduced the spin–spin relaxation time (T_2_) to enhance magnetic resonance imaging (MRI) contrast and improve the quality of MRIs (Figure 8c). Moreover, the low cytotoxicity and good biocompatibility of this hydrogel were further proved in this research. With a good self-healing ability, injectability, biosafety, magnetic property, and thermal responsiveness in alternating magnetic fields, the hydrogel had great potential in the application of MRI contrast agents and implantable biomedical materials. This work demonstrates the power of the KF reaction in the synthesis of functional polymers and its further use in the development of smart materials.

## 4. Flame Retardants

**Figure 9:**
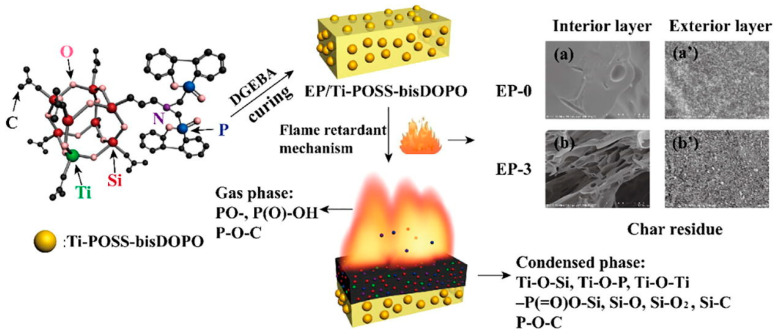
A novel ternary metal–silicon–phosphorus intramolecular hybrid flame retardant based on Ti-embedded and DOPO-functionalized POSS for the modification of EP. (**a**) The interior layer of EP-0. (**b**) The interior layer of EP-3. (**a’**) The exterior layer of EP-0. (**b’**) The exterior layer of EP-3. The Reprinted with permission from ref. [51]. Copyright 2019 Elsevier B.V. All rights reserved.

Many polymer materials are severely limited in their practical applications due to their flammability [48,49]. Multiple flame retardants have been developed to improve flame retardancy in polymers. Among them, phosphorus-based flame retardants have great potential for replacing traditional halogenated flame retardants for enhancing the flame retardancy of polymers due to their high flame retardant efficiency, low toxicity, and good compatibility [50]. Recently, the KF reaction has attracted attention in the field of polymer flame retardants for the efficient introduction of P elements through α-aminophosphonate structures. In 2019, Dai et al. successfully synthesized a new metal–silicon–phosphorus intramolecular hybrid as a flame retardant to improve the flame retardant properties of epoxy resin (EP) (Figure 9) [51]. In this research, one Ti atom was embedded into the polyhedral oligomeric silsesquioxanes (POSS) cage network, and two dibenz[C,E] [1,2] oxaphosphorin6-oxide (DOPO) units with flame retardant properties were grafted to the aminopropyl groups from POSS via the KF reaction. The metal–organic–inorganic hybrid (Ti-POSS-bisDOPO) was herein obtained and incorporated into EP as a flame retardant. The EP with Ti-POSS-bisDOPO exhibited a good performance in flame retardancy, where the P elements in DOPO produced P free radicals to interrupt the chain reactions of combustion and formed a dense and phosphorus-rich carbon layer (dehydration effect) to block the heat. This research paves the way for the application of the phosphorus-containing structure generated by the KF reaction in halogen-free phosphorus flame retardants and further taps into the potential of the KF reaction in polymer chemistry and material science.

**Figure 10:**
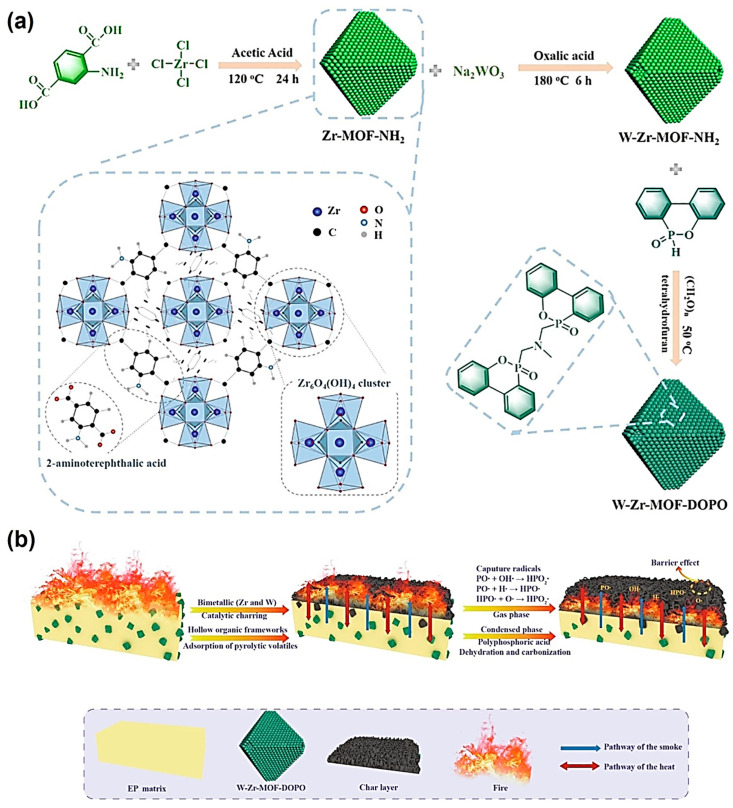
(**a**) Preparation process of the organophosphorus-modified hollow bimetallic organic framework. (**b**) Flame retardancy mechanisms for the EP mixed with the organic frameworks. Reprinted with permission from ref. [27]. Copyright 2021 Elsevier B.V. All rights reserved.

In addition to the abovementioned work, Dai et al. applied metal-organic frameworks (MOFs) instead of POSS for blending with EP to prepare polymer composites with high flame retardancy and smoke suppression capabilities [27]. The preparation process of the organic framework is shown in Figure 10a, and the KF reaction was used for grafting organophosphorus onto the organic framework. The final product improved the flame retardancy of EP through a series of mechanisms to inhibit the generation of toxic smoke and heat during combustion (Figure 10b). By combining hollow MOF with polymer materials, this work enhanced their fire safety for the first time, further promoting the application of phosphorus-containing structures produced by KF reactions in flame retardants.

## 5. Bioimaging Probes

**Figure 11:**
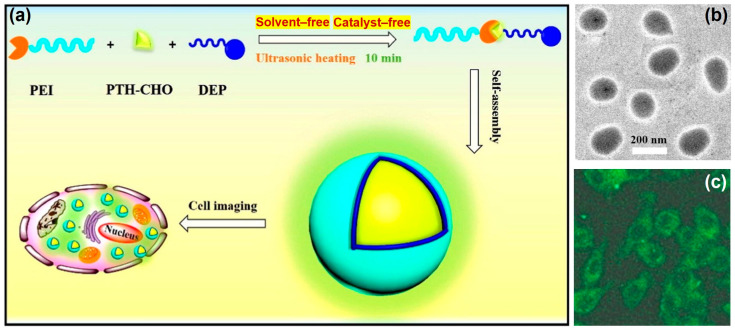
(**a**) Preparation process and cell imaging properties of the AIE-active FNPs. (**b**) TEM image of the AIE-active FNPs dispersed in water. (**c**) Fluorescent imaging under excitation with a 405 nm laser. Reprinted with permission from ref. [52]. Copyright 2016 Elsevier B.V. All rights reserved.

Besides generating functional α-aminophosphonate structures, the KF reaction can also serve as an efficient and powerful coupling tool. By combining the KF reaction with fluorescent molecules, such as fluorescent dye and aggregation-induced emission (AIE), researcher can design and fabricate commercially available polymers into bioimaging probes in a straightforward manner. In 2016, Zhang et al. first reported a novel “one pot” strategy for the ultrasound-assisted, catalyst-free, and solvent-free preparation of AIE-active fluorescent organic nanoparticles (FNPs) via the KF reaction [52]. Nanoparticles with AIE activity that self-assembled in an aqueous solution were efficiently prepared by ultrasonically heating polyetherimide (PEI), an AIE part (PTH-CHO), and diethyl phosphite (DEP) for only 10 min (Figure 11a). The hydrophobic PTH-CHO was encapsulated by hydrophilic PEI to form nanoparticle assemblies (~200 nm) with good dispersibility in water (Figure 11b). Furthermore, these AIE-active FNPs exhibited low cytotoxicity and emitted a strong green fluorescence under 405 nm laser excitation for cell imaging (Figure 11c). This study demonstrated the great potential of the KF reaction for the synthesis of polymeric bioimaging probes.

**Figure 12:**
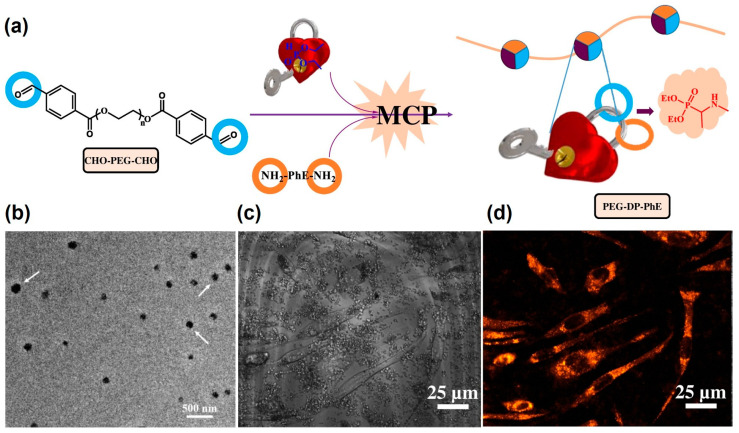
(**a**) Preparation of PEG-DP-PhE FPNs by MCP based on a microwave-assisted KF reaction. (**b**) TEM image of the PEG-DP-PhE FPNs. (**c**) Bright field images of L929 cells co-cultured with the PEG-DP-PhE FPNs. (**d**) Fluorescent imaging under excitation with a 458 nm laser. Reprinted with permission from ref. [53]. Copyright 2017 Elsevier B.V. All rights reserved.

As a continuation of this research, Zhang et al. further developed a solvent-free, catalyst-free microwave-assisted multicomponent tandem polymerization (MCP) technology for the construction of AIE-active FPNs [53]. In this study, the amphiphilic polymer (PEG-DP-PhE) was prepared by coupling a diamino AIE-reactive dye (H_2_N-PhE-NH_2_) with dialdehyde-terminated polyethylene glycol (CHO-PEG-CHO) through the KF reaction (Figure 12a). The PEG-DP-PhE self-assembled in an aqueous solution to form a regular spherical particle (~100 nm) with a double-layered structure (Figure 12b). After being co-cultured with L929 cells, a good cell morphology was observed under a bright field (Figure 12c), and a strong orange-yellow fluorescence signal was observed under 458 nm laser excitation (Figure 12d), indicating the good biocompatibility and bioimaging capabilities of the PEG-DP-PhE FPNs. This work presented a new method to simply prepare nanoparticles and further demonstrated the advancement of the KF reaction in the efficient preparation of AIE-active nanoparticles for bioimaging probes.

**Figure 13:**
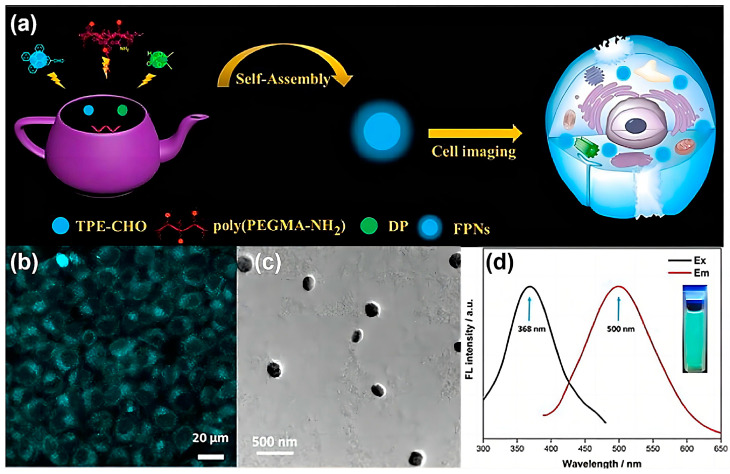
(**a**) Preparation of the PEGMA-TPE FPNs through an efficient post-modification method through a microwave-assisted KF reaction for biological imaging applications. (**b**) Fluorescent imaging under excitation with a 405 nm laser. (**c**) TEM image of the PEGMA-TPE FPNs. (**d**) Excitation (Ex) and emission (Em) fluorescent spectra of the PEGMA-TPE FPNs dispersed in water. Ex = 368 nm, Em = 500 nm. Reprinted with permission from ref. [54]. Copyright 2017 Elsevier B.V. All rights reserved.

The post-modification of polymers was also developed to prepare AIE-active FPNs. Based on their previous work, Zhang et al. proposed a simple strategy for the preparation of AIE-active FPNs through an efficient post-modification method through a microwave-assisted KF reaction (Figure 13a) [54]. Taking advantage of the high efficiency of the KF reaction, poly (PEGMA-NH_2_) was post-modified with an AIE-active dye (TPE-CHO) and diethyl phosphate (DP) to prepare PEGMA-TPE within just 5 min under microwave irradiation. The PEGMA-TPE FPNs were easily obtained by dispersing PEGMA-TPE in an aqueous solution. The PEGMA-TPE FPNs were distributed in the cytoplasm after endocytosis, and strong fluorescence signals were observed under 405 nm laser excitation (Figure 13b). Due to the strong hydrophobic interaction and π-π interaction between TPE-CHO, the PEGMA-TPE FPNs self-assembled into uniform spherical particles with diameters between 100 and 200 nm (Figure 13c). The maximum emission wavelength of the PEGMA-TPE FPNs was located at 500 nm, and the optimal excitation wavelength was located at 368 nm. Consistent with the fluorescence spectrum, a uniform and strong green fluorescence was observed after irradiation of the PEGMA-TPE FPNs with an ultraviolet lamp at 365 nm (Figure 13d). With their small particle size, good water dispersibility, strong fluorescence, and low cytotoxicity, the AIE-active PEGMA-TPE FPNs were endowed with great potential in biomedical applications. This work demonstrates the simplicity and efficiency of the microwave-assisted KF reaction in the preparation of AIE-active FPNs by means of polymer modifications, avoiding complex separation and purification.

## 6. Other Applications

**Figure 14:**
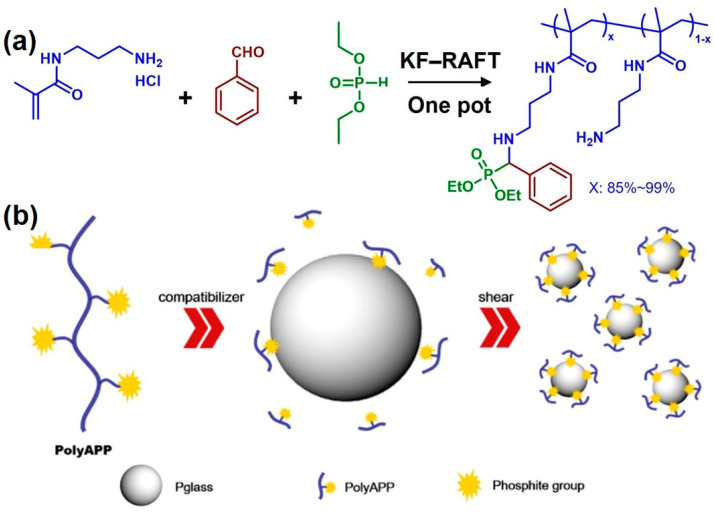
(**a**) Preparation of polyApp through “one pot” KF-RAFT polymerization. (**b**) Mechanisms for compatibility enhancement of Pglass/polymer composites by adding polyAPP. Reprinted with permission from ref. [55]. Copyright 2018 Elsevier Ltd. All rights reserved.

In recent years, some emerging applications of functional polymers prepared via the KF reaction have also been reported. In 2018, Fu et al. developed a newly synthesized compatibilizer (polyApp) for tin fluorophosphate glass (Pglass)/polymer composites through “one pot” KF-reversible addition fragmentation chain transfer (RAFT) polymerization (Figure 14a) [55]. Amine, benzaldehyde, and diethyl phosphite reacted by means of the KF reaction to form α-aminophosphonate (APP) structures in the side chains of the polymer. The polymer chains interacted with the polymer matrix, and APP interacted with Pglass, which made polyAPP act as an adhesive at the interface between the Pglass and the polymer matrix. Compared with traditional compatibilizers, polyAPP enhanced the interfacial interaction between Pglass and maleic anhydride-grafted polypropylene (MA-PP) and decreased the dispersed phase size of Pglass in the polymer matrix (Figure 14b). Meanwhile, the mechanical properties of the Pglass/polymer composites were also improved, due to the enhanced interaction between the Pglass and the polymer matrix. In comparison with the original MA-PP, the tensile strength and modulus of the composites were increased by 22% and 33%, respectively. This study broadened the application boundary of functional polymers prepared through the KF reaction and provided a new method for the preparation and modification of high-performance Pglass/polymer composites and a series of functional polymer composites.

**Figure 15:**
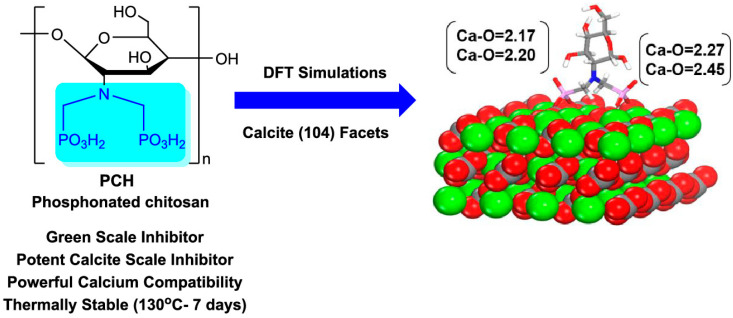
Structure of the PCH as an environmentally friendly scale inhibitor and the DFT simulation of descaling mechanisms. Reprinted with permission from ref. [56]. Copyright 2021 The Authors. Published by the American Chemical Society. This publication is licensed under CC-BY 4.0. All rights reserved.

In 2021, Mady et al. synthesized phosphonated chitosan (PCH) as an environmentally friendly scale inhibitor through the KF reaction (Figure 15) [56]. Due to the metal chelating ability of the α-aminophosphonate structure produced by the KF reaction, PCH showed good performance as a calcium carbonate scale inhibitor, similar to some commercial scale inhibitors. Meanwhile, PCH showed an excellent calcium compatibility performance, and the performance was not lost even after thermal aging in an anaerobic aqueous solution (5 wt%) for 1 week at 130 °C. These results indicated that PCH was an excellent descaling agent for inhibiting calcium carbonate, with good calcium ion compatibility and thermal stability. Density functional theory (DFT) and molecular dynamics (MD) simulations were carried out to reveal the strong interaction between PCH and mineral surfaces and the descaling mechanisms. This work showed the application of the KF reaction in the synthesis of environmentally friendly oilfield scale inhibitors and provided a new insight for functional polymers prepared via the KF reaction.

## 7. Perspectives and Conclusions

Over the last few years, the KF reaction has shown its power in the synthesis of functional polymers. Through its unique α-aminophosphonate structures, the KF reaction has enriched polymers with useful functions such as metal chelation and flame retardancy abilities. Owing to its high reaction efficiency and abundant reactant choices, the KF reaction has also proven to be an effective tool for polymer modification and coupling, allowing the design of new multicomponent functional polymers with more complex functional motifs.

In this mini-review, we systematically summarized functional polymers based on the KF reaction in different applications, such as acting as heavy metal adsorbents, multifunctional hydrogels, flame retardants, and bioimaging probes. We also discussed some emerging applications of functional polymers prepared via the KF reaction. We believe that the excellent performance of these KF polymers will play an important part in polymer synthesis, material science, biomedicine, and other disciplines. For example, (i) the further exploration of KF polymers in the synthesis of various polymer backbones and side chains will promote the generation of new functional polymer structures. (ii) The mining of more adaptive functional substrates for the KF reaction will further enrich the functions of polymers. (iii) Moreover, the combination of the KF reaction with more disciplines to prepare new polymers will also promote more practical applications.

In fact, there is still much room for using the KF reaction to explore new functional polymers. We only listed several possible research directions, but the options are not limited to these. (1) The development of polymers with different topological structures, such as hyperbranched polymers containing α-aminophosphonate structures as the linking points through polycondensation and hyperbranched polymers with pendent α-aminophosphonate structures through FRP or PPM strategies, is one of such directions. (2) Another is the development of polymers with precise structures through the KF reaction, such as dendrimers with α-aminophosphonate structures as the branch points or sequence-controlled polycondensates. (3) The development of new functional polymers based on the new properties of α-aminophosphonate structures (metal chelating, antibacterial, antifungal, anticancer, etc.) is yet another direction for future research. Therefore, we look forward to the further development of the KF reaction and its novel applications across multiple disciplines.

## Data Availability

Not applicable.
